# Supplementary material for: Axonal G3BP1 stress granule protein limits axonal mRNA translation and nerve regeneration
Source: Nat Commun. 2018 Aug 22;9:3358. doi: 10.1038/s41467-018-05647-x (PMC6105716; doi:10.1038/s41467-018-05647-x)
Supplement: Supplementary file 1 — Supplementary Information [file 41467_2018_5647_MOESM1_ESM.pdf]

Axonal G3BP1 stress granule protein limits axonal mRNA translation and nerve regeneration

Sahoo et al.

Supplementary Information:

- Supplementary Figures 1-9

Supplementary Figure 1

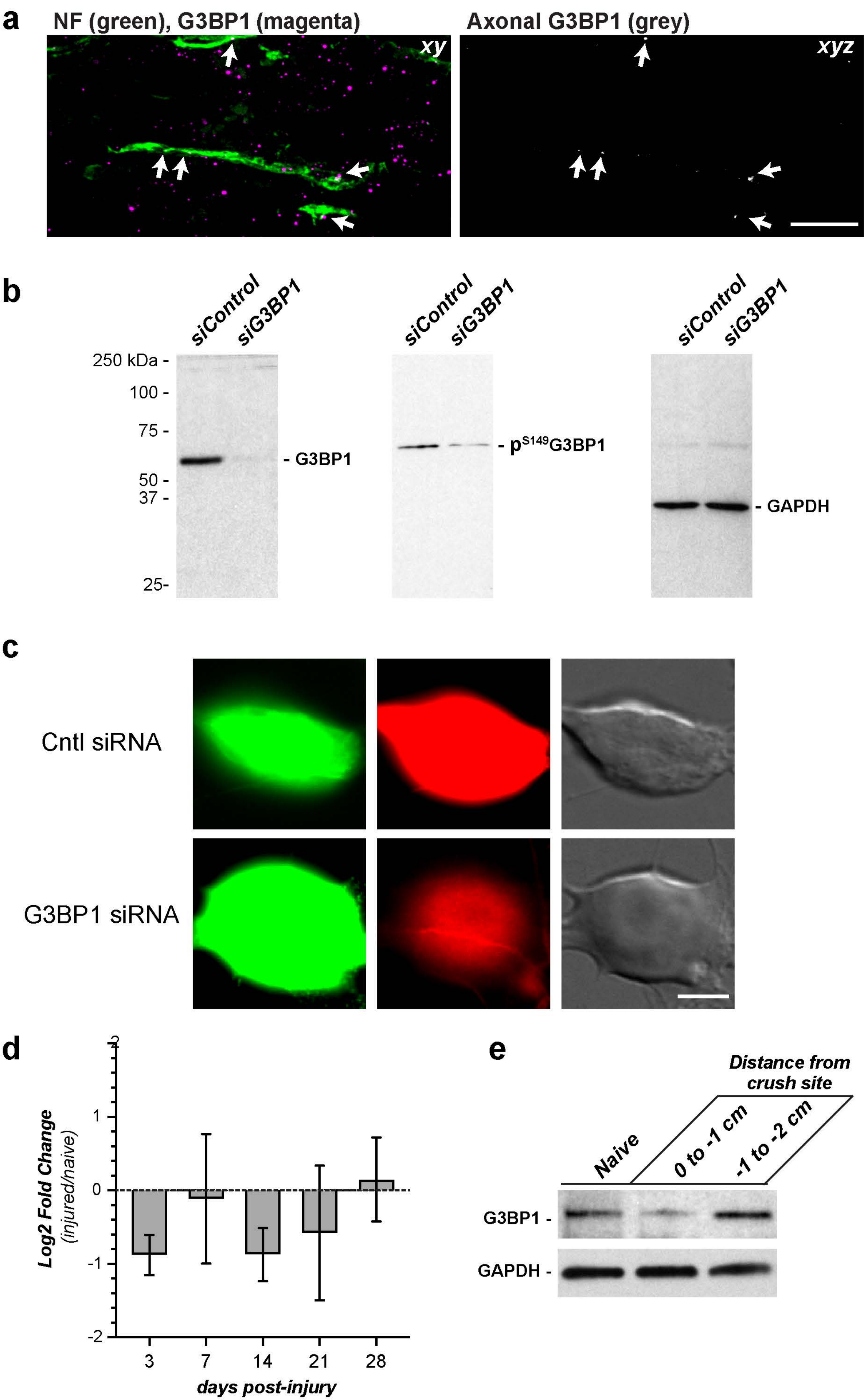

<u>Supplementary Figure 1</u>: ***G3BP1 is present in peripheral nerves.***
a, Representative images of most distal regenerating axons (7 d post-sciatic nerve crush injury) are shown. Left panel shows G3BP1 merged with NF signals in XY projection. Right panel shows XYZ projection of G3BP1 signals overlapping with NF (i.e., axonal G3BP1) across individual optical planes of the Z stack [scale bar = 10 µm].
b, Immunoblots showing single bands at anticipated molecular weights for anti-(pan)G3BP1 and -G3BP1^{PS149} from control and G3BP1 siRNA transfected DRG cultures.
c, Representative exposure matched images for anti-G3BP1^{PS149}, anti-NF, and DIC in control (Cntl) vs. G3BP1 siRNA transfected DRG cultures [scale bar = 10 µm].
d, Median log2 fold-change (log2FC) values for exoplasm G3BP1 abundance (quantified as sum of MS2 peak areas by PRM) compared to naïve nerve in axoplasm prepared from approx. 3 cm nerve segments immediately proximal to crush site at indicated durations after injury. There are modest, but not statistically significant changes in G3BP1 spectral counts following injury.
d, Representative immunoblots for G3BP1 and GAPDH control are shown for axoplasm harvested from 1 cm segments of sciatic nerve taken at 0 to -1 and -1 to -2 cm proximal to injury site compared to a corresponding segment of uninjured sciatic nerve.

Supplementary Figure 2

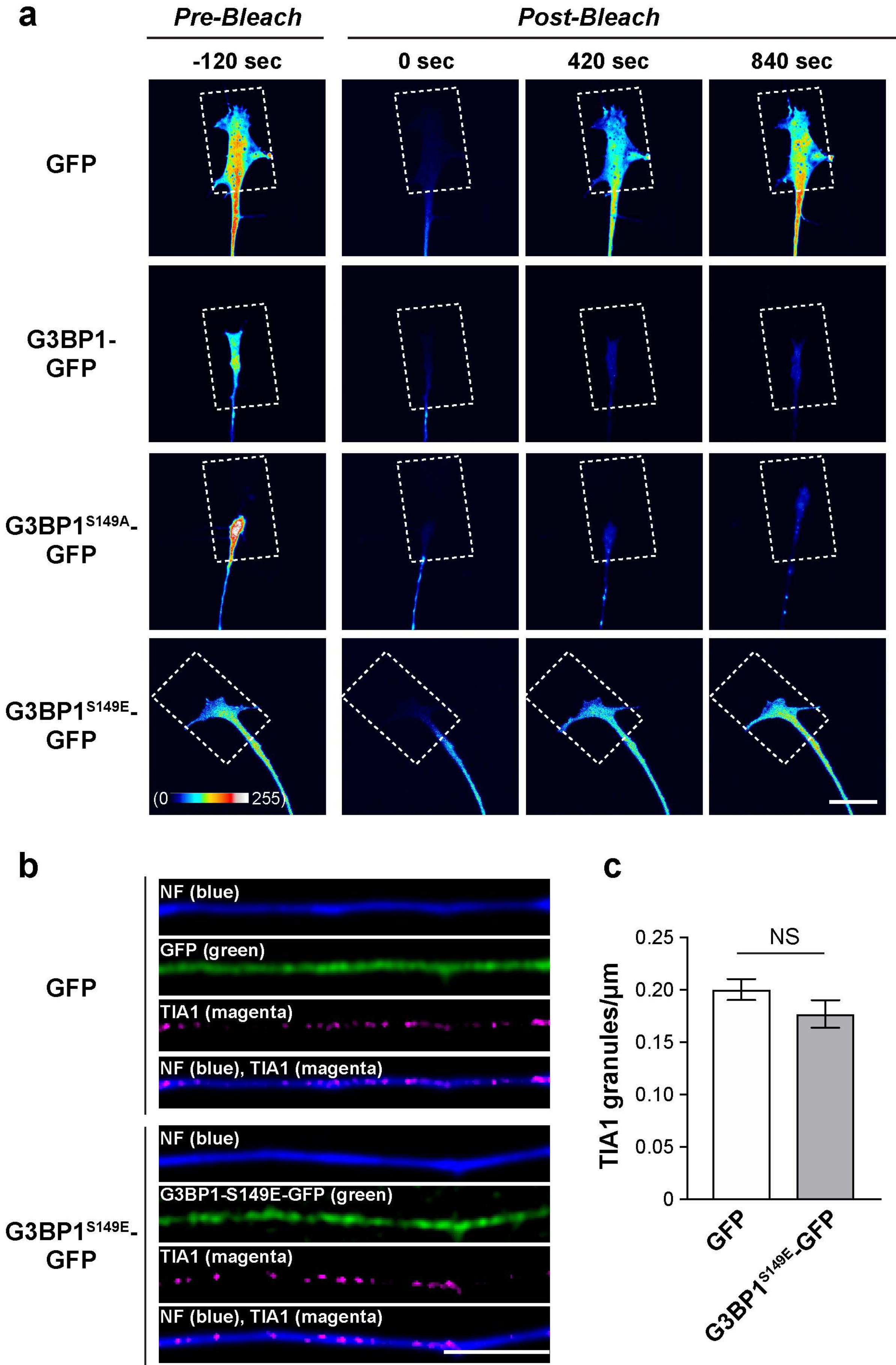

Supplementary Figure 2: ***Expression of phosphmimetic G3BP1 in DRG cultures.***
a, Representative FRAP image sequences for DRG neurons transfected with GFP, G3BP1-GFP, G3BP1^{S149A}-GFP, or G3BP1^{S149E}-GFP (36 h post-transfection) are shown. Boxed regions represent the photobleached ROIs. Refer to **Fig. 2c** for quantitative data [Scale bar = 20 µm].
b, Representative immunofluorsence signals for TIA1, GFP, and NF in axons of DRG neurons transfected with eGFP vs. G3BP1^{S149E}-GFP [scale bar = 10 µm].
c, Axonal TIA1 aggregates are not altered by introduction of the phosphomimetic G3BP1. Values are average ± SEM (N ≥ 34 axons over 3 repetitions; NS = not significant by one-way ANOVA with Tukey HSD post-hoc).

Supplementary Figure 3

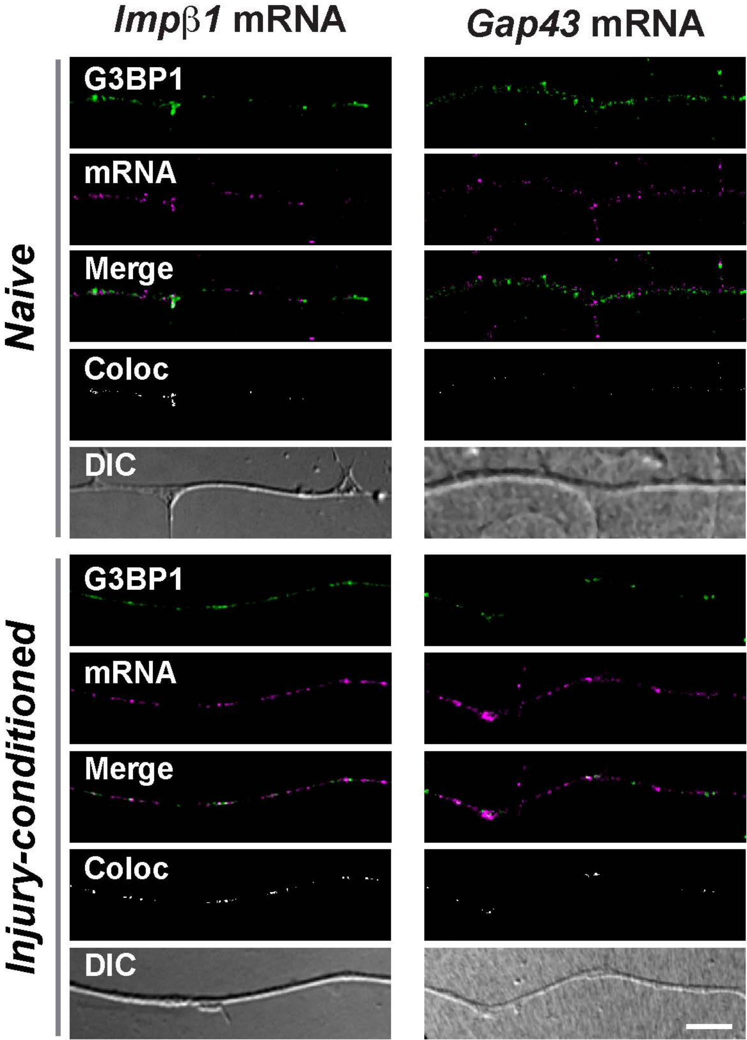

__Supplementary Figure 3__: ***Axonal G3BP1 colocalizes with Impβ1 but not Gap43 mRNAs.*** Representative FISH/immunofluorescence images of axons from DRG cultures for *Impβ1* and *Gap43* mRNAs, G3BP1, and NF are shown. Colocalized signals for G3BP1 and the indicated mRNAs (Coloc) were generated with the ImageJ Colocalization plug-in [scale bar = 5 μm].

Supplementary Figure 4

a

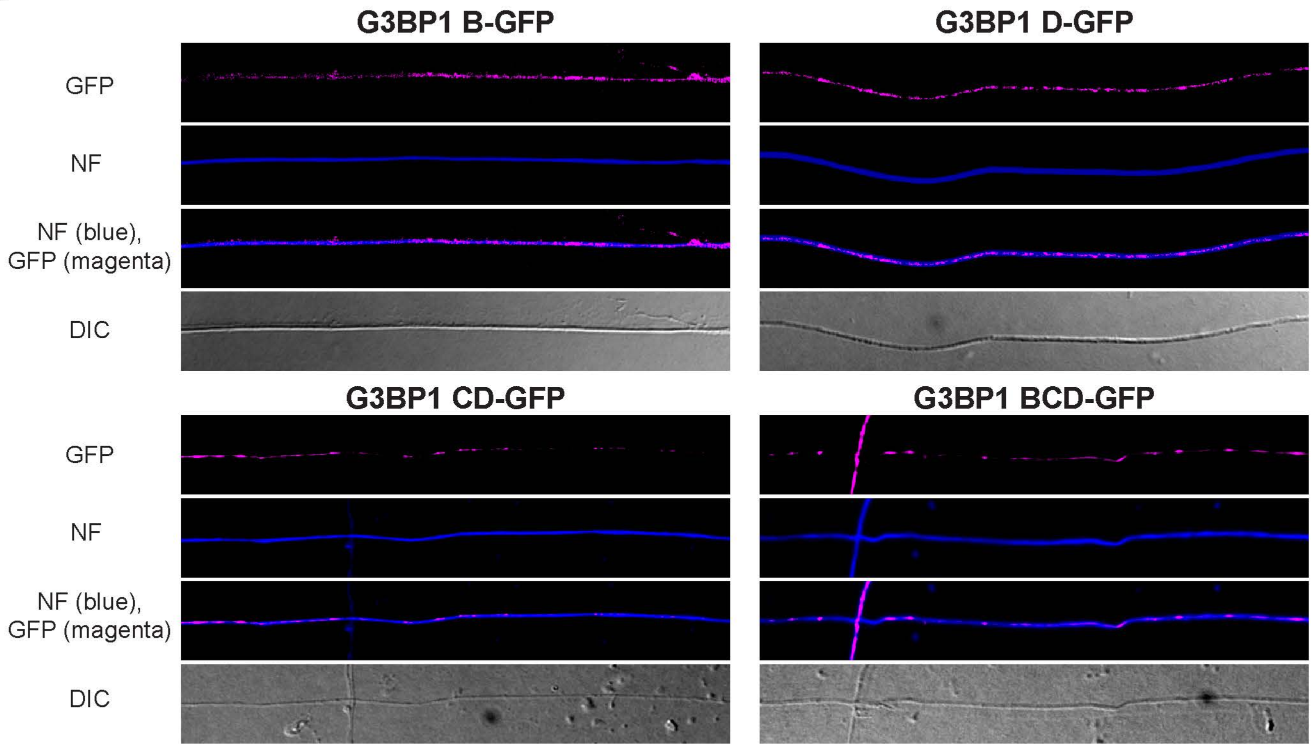

b

	***Total axon length** (µm)*	***Longest axon** (µm)*	***Axons/ neuron***
GFP	3522 ± 259	649 ± 36	7.2 ± 0.32
G3BP1-GFP (ABCD domain-GFP)	3993 ± 322 *[NS]*	**745 ± 46** *[p ≤ 0.005]*	**6.22 ± 0.28** *[p ≤ 0.05]*
G3BP1^{S149A}-GFP	3652 ± 351 *[NS]*	745 ± 46 *[NS]*	6.3 ± 0.25 *[NS]*
G3BP1^{S149E}-GFP	4026 ± 377 *[NS]*	760 ± 46 *[NS]*	7 ± 0.47 *[NS]*
A domain-GFP	**4573 ± 329** *[p ≤ 0.05]*	**801 ± 44** *[p ≤ 0.01]*	7.2 ± 0.31 *[NS]*
B domain-GFP	**6396 ± 726** *[p ≤ 0.0005]*	**1031 ± 55** *[p ≤ 0.0001]*	**6.1 ± 0.30** *[p ≤ 0.05]*
C domain-GFP	3893 ± 315 *[NS]*	**792 ± 40** *[p ≤ 0.01]*	7.1 ± 0.31 *[NS]*
D domain-GFP	**2329 ± 219** *[p ≤ 0.001]*	**456 ± 35** *[p ≤ 0.0005]*	7.3 ± 0.38 *[NS]*
CD domain-GFP	**2144 ± 165** *[p ≤ 0.0001]*	**517 ± 27** *[p ≤ 0.005]*	**6.0 ± 0.28** *[p ≤ 0.01]*
BCD domain-GFP	**5675 ± 327** *[p ≤ 0.0001]*	**910 ± 48** *[p ≤ 0.0001]*	7.2 ± 0.42 *[NS]*

c

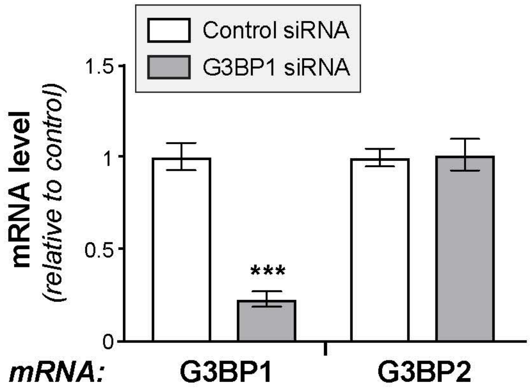

d

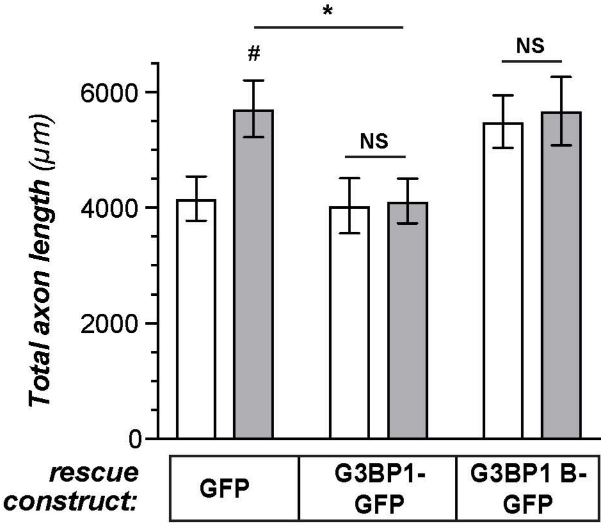

<u>Supplementary Figure 4</u>: ***G3BP1 B domain increases axon growth.***
a, Representative images of distal axons of naïve DRG neurons were transfected with G3BP1 B domain-GFP, D domain-GFP, CD domain-GFP, or BCD domain-GFP are shown. Images were captured at > 200 µm away from the cell body show [scale bar = 20 µm].
b, Axon growth values for DRG neurons transfected with indicated expression constructs are shown as mean ± SEM, with blue font indicating significant values compared to GFP control. Expression of the G3BP1 B domain caused the greatest increase in axon growth while the D domain decreased axon growth (N ≥ 100 neurons each over 3 culture preparations; p values by one-way ANOVA with Tukey HSD post-hoc).
c, RT-ddPCR analyses of G3BP1 and G3BP2 mRNA levels in DRGs transfected with control and G3BP1 siRNAs are shown. GAPDH mRNA level was used for normalization (N = 3 culture preparations; *** p ≤ 0.0005 vs. control siRNA by Student's t-test).
d, Axon length for control vs. G3BP1 depleted DRG cultures that were co-transfected with GFP, siRNA-resistent G3BP1-GFP, or G3BP1 B domain-GFP are shown as mean ± SEM. The siRNA-resistent G3BP1-GFP completely reverses the growth-promoting effect of G3BP1 depletion, but the G3BP1 B domains does not result in further increase in axon length above G3BP1-depletion (N ≥ 51 neurons over 3 repetitions; * p ≤ 0.01 between indicated groups and # p ≤ 0.05 for indicated sample vs. control siRNA + GFP by one-way ANOVA with Tukey HSD post-hoc).

Supplementary Figure 5

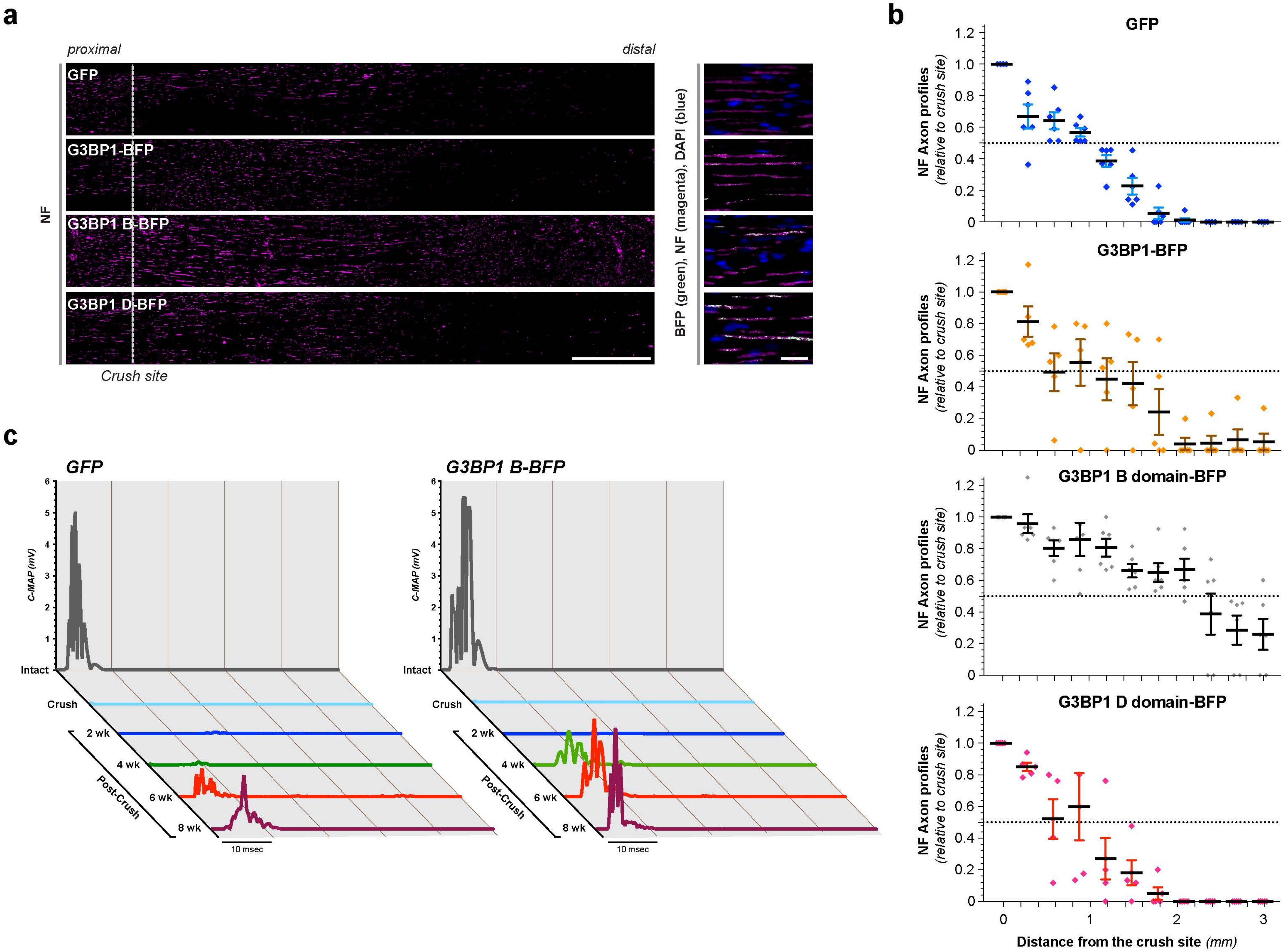

<u>Supplementary Figure 5</u>: ***G3BP1 B domain expression increases PNS axon regeneration in vivo.***
a, Representative, exposure-matched confocal images of crushed sciatic nerves of adult rats transduced with AAV5 encoding the G3BP1-BFP, G3BP1 B domain-BFP, G3BP1 D domain-BFP or GFP are shown (7 d post-nerve crush; 14 d post-transduction). NF signals are shown with proximal on left, distal on right, and crush site indicated. The right hand column shows high magnification single optical planes with intra-axonal signals for G3BP1-BFP, B domain-BFP, and D domain-BFP proteins proximal to the injury site [Scale bars = 500 µm for left column and 25 µm for right column].
b, Graphs for individual data points for the nerve regeneration analyses from **Fig. 4c** are shown. Black horizontal lines indicate mean values with SEM shown as colored vertical capped lines.
c, Representative C-MAP tracings for left and right lateral gastrocnemius following sciatic nerve stimulations are shown for 0-8 wk following crush injury. Animal was transduced with AAV5 encoding GFP and B domain-BFP at 7 d prior to crush injury (see **Fig. 4d** for quantitation).

Supplementary Figure 6

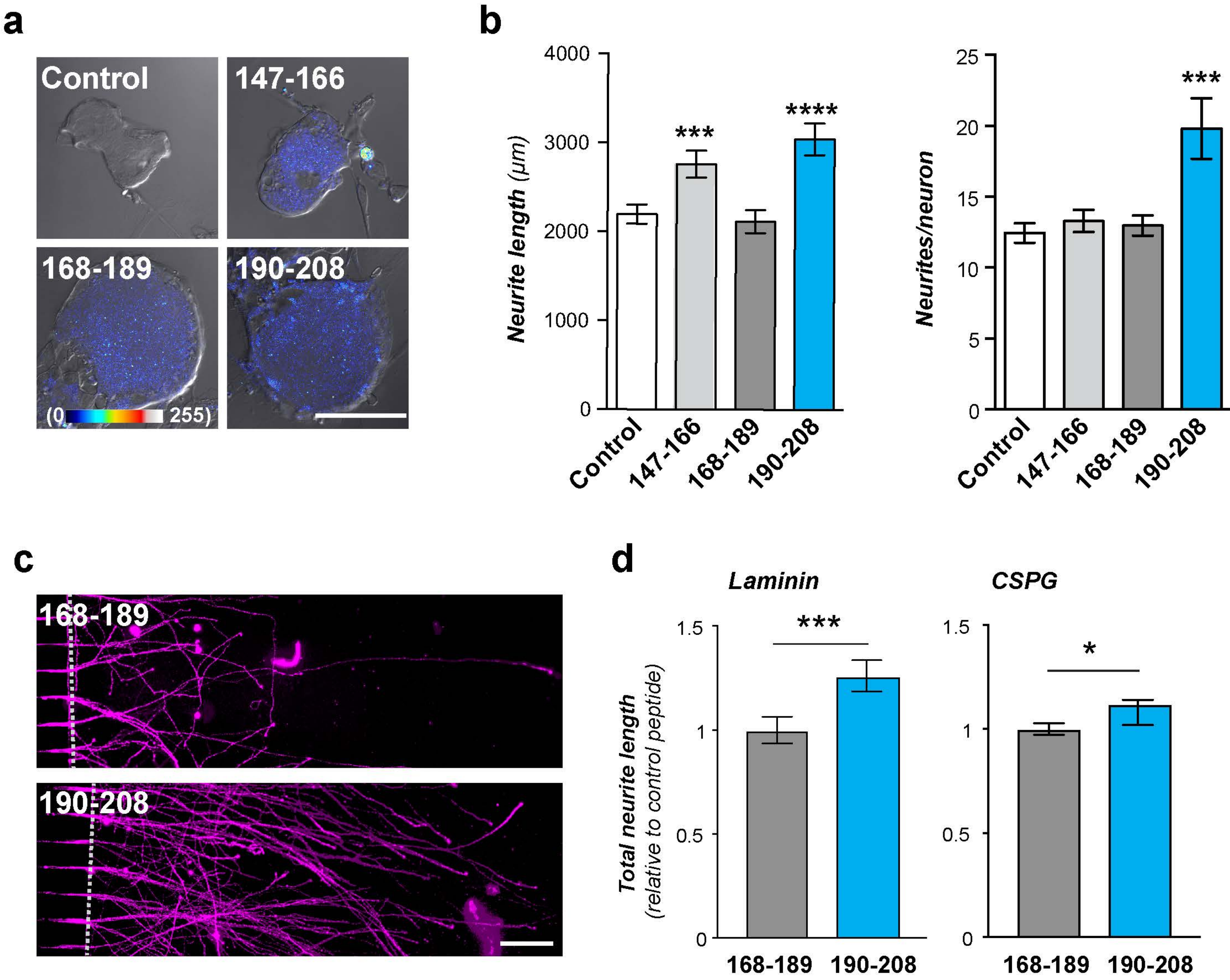

Supplementary Figure 6: ***G3BP1 B domain inhibits SG formation and up-regulates axonal mRNA translation.***
a, Representative images of dansyl chloride fluorescence of internalized peptides in neuronal cell bodies of DRG cultures at 30 min post-treatment [scale bar = 20 µm].
b, Neurite outgrowth analyses for dissociated DRG neurons treated with cell permeable G3BP1 peptides immediately after plating is shown. 190-208 peptide significantly increases both total neurite length and neurites/neuron, while 147-166 peptide only increases neurite length ($N \geq 254$ neurons each over 3 repetitions; *** = $p \leq 0.005$; **** = $p \leq 0.001$ by one-way ANOVA with Tukey HSD post-hoc).
c, Representative images of axonal compartment of microfluidic culture device with cortical neurons stained for tau at DIV 6. 10 µM 168-189 and 190-208 peptides were added only to the axons at DIV 3; dashed line indicates the beginning of axonal compartment with axons exiting grooves of the barrier. Quantification of axonal growth for these cultures is shown in **Fig. 4e** [scale bar = 500 µm].
d, Human motor neurons generated from differentiated human iPSCs exposed to 10 µM 190-208 peptide show significantly increased axon growth on both laminin and CSPG substrates compared to 160-189 peptide treated neurons ($N = 4$; * $p \leq 0.05$, *** $p \leq 0.001$ by Student's t-test).

Supplementary Figure 7

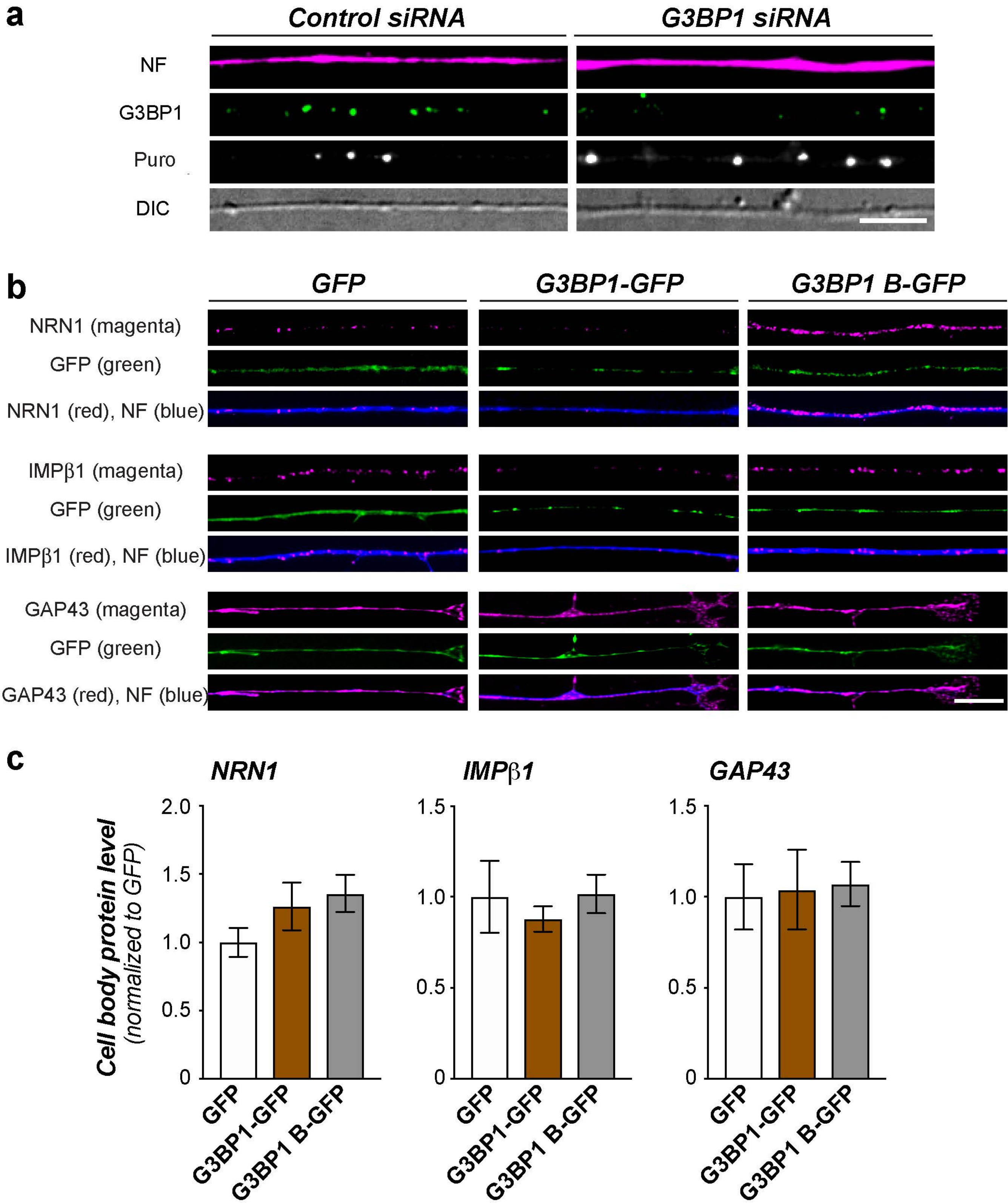

<u>Supplementary Figure 7</u>: ***G3BP1 B domain inhibits G3BP1 function.***

a, Representative images for puromycin incorporation in axons of DRG neurons transfected with control vs. G3BP1 siRNAs are shown [Scale bar = 5 µm].

b, Representative images of DRG axons for NRN1, IMPβ1, and GAP43 proteins after transfection with GFP, G3BP1-GFP or G3BP1 B domain-GFP (see **Fig. 5d** for quantitation) [Scale bar = 10 µm].

c, Quantitation of cell NRN1, IMPβ1, and GAP43 immunoreactivity in cell bodies of DRG neurons transfected as in b are shown. There are no statistical differences between the GFP, G3BP1-GFP or G3BP1 B domain-GFP expressing DRG neurons.

Supplementary Figure 8

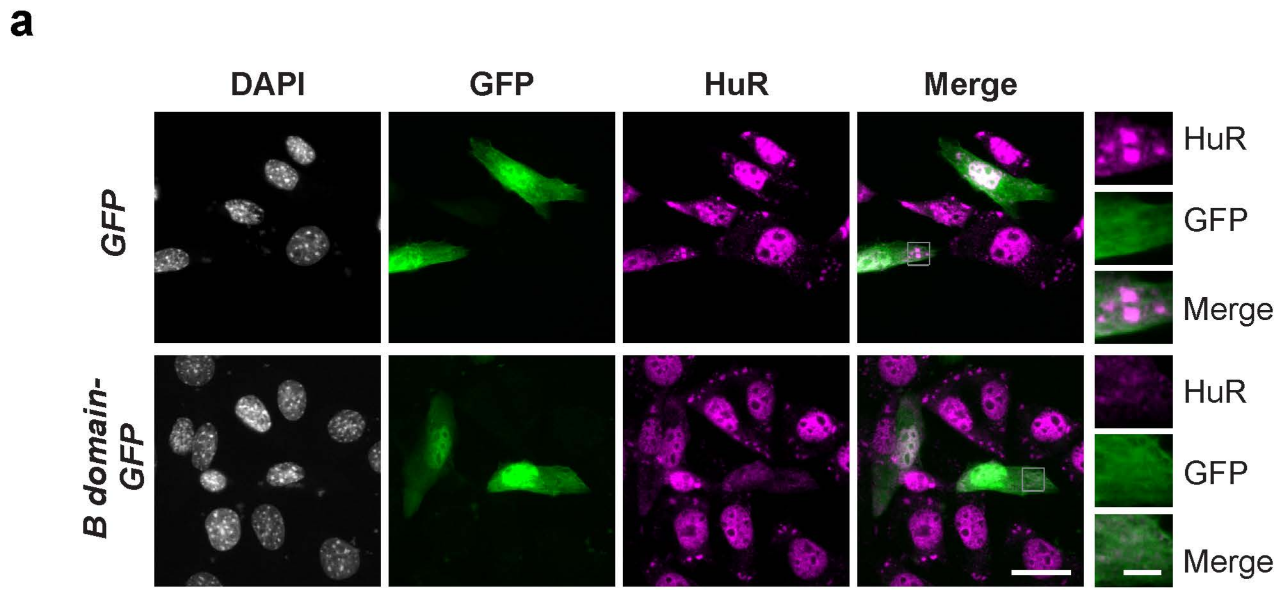

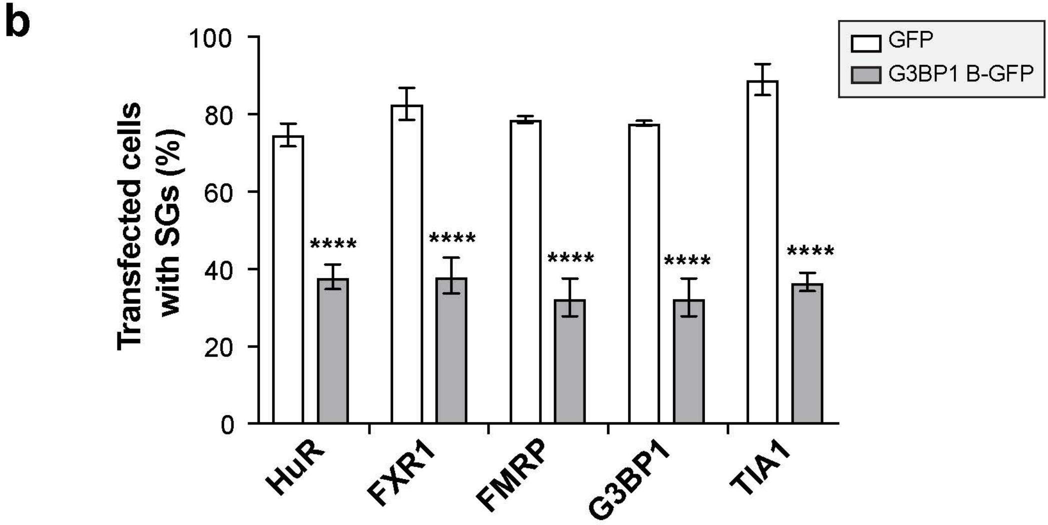

Supplementary Figure 8: ***G3BP1 B domain blocks induced SG formation.***
a, Representative images of HuR immunoreactivity in NIH-3T3 cells that were transfected GFP vs. G3BP1 B domain-GFP are shown after 30 min treatment with sodium arsenite (0.5 mM). The GFP-transfected cells show decrease in cytoplasmic SGs with expression of G3BP1 B domain-GFP. The regions in boxes in the merge column are shown at high magnification in the last column as indicated [Scale bars = 20 µm for first 4 columns and 5 µm for right column].
b, Quantification SGs in the transfected NIH-3T3 cells from A are shown based on the indicated immunostaining. Expression of the G3BP1 B domain significantly decreases the percentage of cells with SG (N = 3 replicates with ≥ 100 transfected cells were analyzed per replicate; **** $p \leq 0.0001$ by Student's t-test).

Supplementary Figure 9

Supplementary Figure 1d

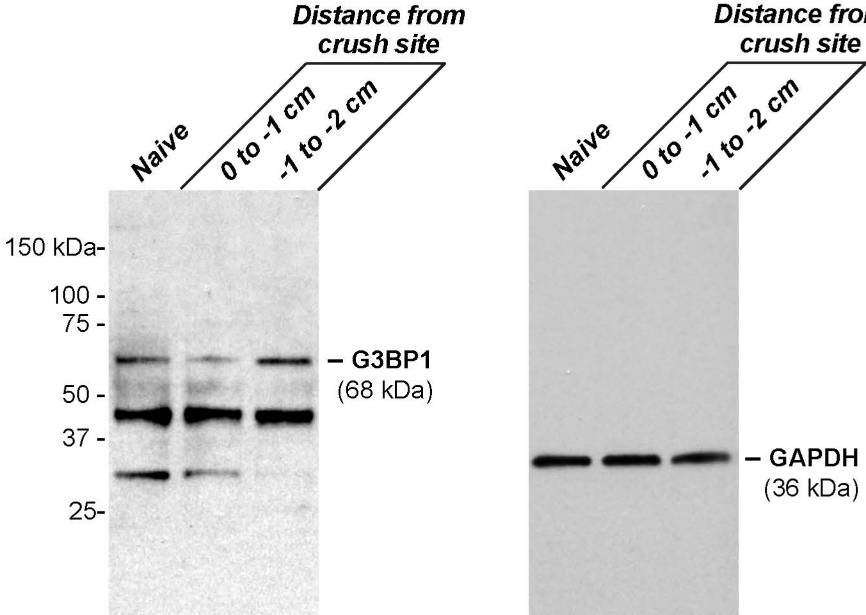

Figure 3h

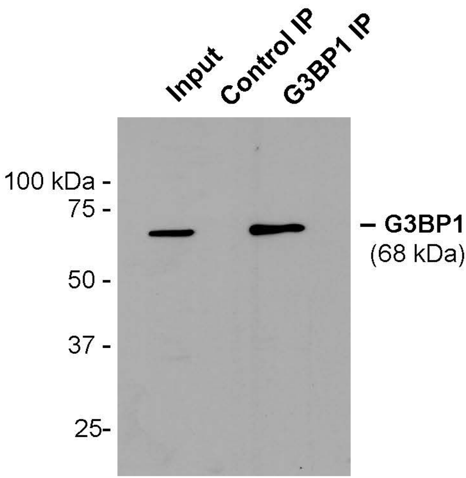

<u>Supplementary Figure 9</u>:
Full gel blot data for Suppl. Fig. S1c and Fig. 3h. Band at predicted molecular weights of G3BP1 and GAPDH are indicated. The additional bands in G3BP1 immunoblots are only seen on axoplasm preparations and not in other protein lysate preparations.
