# Supplementary material for: Axonal G3BP1 stress granule protein limits axonal mRNA translation and nerve regeneration
Source: Nat Commun. 2018 Aug 22;9:3358. doi: 10.1038/s41467-018-05647-x (PMC6105716; doi:10.1038/s41467-018-05647-x)
Supplement: Supplementary file 3 — Description of Additional Supplementary Files [file 41467_2018_5647_MOESM3_ESM.pdf]

Description of Additional Supplementary Files

File Name: Supplementary Movie 1

Description: Representative FRAP videos for GFP^{MYR}5'/3'nrn1 in axons of DRG neurons co-transfected with BFP (left) or G3BP1-BFP (right) are shown. Boxed regions represent the photobleached ROIs; refer to **Fig. 3e** for quantitation. Frames prior to 0 sec time point shows the pre-bleach fluorescence, and the post-bleach sequence begins with the frame for 0 sec time point

File Name: Supplementary Movie 2

Description: Representative FRAP videos for GFP^{MYR}5'/3'impβ1 in axons of DRG neurons co-transfected with BFP (left) or G3BP1-BFP (right) are shown. Boxed regions represent the photobleached ROIs; refer to **Fig. 3f** for quantitation. Frames prior to 0 sec time point shows the pre-bleach fluorescence, and the post-bleach sequence begins with the frame for 0 sec time point.

File Name: Supplementary Movie 3

Description: Representative FRAP videos for mCh^{MYR}5'/3'gap43 in axons of DRG neurons co-transfected with BFP (left) or G3BP1-BFP (right) are shown. Boxed regions represent the photobleached ROIs; refer to **Fig. 3g** for quantitation. Frames prior to 0 sec time point shows the pre-bleach fluorescence, and the post-bleach sequence begins with the frame for 0 sec time point.

File Name: Supplementary Movie 4

Description: Representative live cell imaging sequences of G3BP1-mCh along mid-axon shaft of cultured DRG neurons is shown for control condition (top) or after 15 min exposure to 10 µM cell permeable G3BP1 190-208 peptide (bottom). Velocity for granules was control = 1.38 ± 0.02 and 190-208 treated = 1.48 ± 0.04 µm/sec (P = 0.0001 by students T test); refer to **Fig. 6e-f** for size and density quantitations.
